# The Protective Role of DUSP4 in Retinal Pigment Epithelium Senescence and Degeneration

**DOI:** 10.3390/ijms26083735

**Published:** 2025-04-15

**Authors:** Xiyuan Liu, Zhaoze Ni, Jing Zhang, Xiaoyan Lin, Chenxin Wu, Yuyang Wu, Lingqin Dong, Zongduan Zhang, Zai-Long Chi

**Affiliations:** State Key Laboratory of Eye Health, Eye Hospital of Wenzhou Medical University, Wenzhou 325035, China; lyliuxiyuan@163.com (X.L.); niczea@163.com (Z.N.); wydhcgd1998@163.com (J.Z.); linxiaoyan@eye.ac.cn (X.L.); wu_chenxin2016@163.com (C.W.); wyuyang98@yeah.net (Y.W.); lingqindong@outlook.com (L.D.)

**Keywords:** dual-specificity phosphatase 4, retinal pigment epithelium, senescence, age-related macular degeneration

## Abstract

The retinal pigment epithelium (RPE) serves as a critical guardian of subretinal homeostasis, with its dysfunction implicated in major retinal pathologies, including age-related macular degeneration (AMD) and retinitis pigmentosa. While cellular senescence has emerged as a key driver of RPE degeneration, the molecular mechanisms underlying this process remain incompletely defined. Emerging evidence implicates dual-specificity phosphatase 4 (DUSP4) in cellular stress responses through its antioxidant and anti-inflammatory capacities, yet its role in RPE pathophysiology remains unexplored. Our study reveals a compensatory increase in DUSP4 expression during AMD-associated RPE senescence. To functionally characterize this observation, we knocked down DUSP4 in the RPE of mice via subretinal injection of AAV-shDUSP4. In a sodium iodate-induced dry AMD model, mice with DUSP4 knockdown presented more severe visual impairment than control mice did. To further investigate the molecular mechanism, stable DUSP4-knockout cell lines were constructed via CRISPR/Cas9 technology. The high expression of senescence markers in the DUSP4-knockout cell lines was reversed by DUSP4 overexpression. Furthermore, DUSP4 coordinates the modulation of cell cycle, stress response, and pro-inflammatory signaling by inhibiting the p53, p38, and NF-kB pathways. These findings establish DUSP4 as a multi-functional regulator of RPE senescence. Our work not only elucidates a novel DUSP4-dependent mechanism in AMD pathogenesis but also highlights its therapeutic potential for preserving RPE function in AMD.

## 1. Introduction

Age-related macular degeneration (AMD), a leading cause of irreversible central vision loss in individuals over 55 years of age, has emerged as a critical public health challenge due to global population aging [1]. While dry AMD constitutes approximately 80–90% of cases and progresses gradually via retinal pigment epithelium (RPE) and photoreceptor degeneration, effective therapeutic interventions remain elusive [2]. This therapeutic gap underscores the urgent need for deeper mechanistic insights into AMD pathogenesis to facilitate novel treatment development.

The pathogenesis of dry AMD arises from multifactorial interactions, encompassing both non-modifiable risk factors and modifiable lifestyle factors. Advanced age stands as the predominant independent risk driver [3,4]. Notably, disease prevalence demonstrates a pronounced age-related escalation, with nearly all advanced cases occurring in patients over 60 years of age.

With aging, accumulative molecular, cellular, and physiological damage drives senescence. Senescence is a stress-responsive cellular state marked by irreversible cell cycle arrest, lipofuscin deposition, chronic low-grade inflammation, oxidative stress, and mitochondrial dysfunction and a senescence-associated secretory phenotype (SASP) [5,6]. This process engages multifaceted molecular networks, including cyclin-dependent kinase inhibitors p16^INK4a^ and p21^Cip/WAF1^, regulated through p53/p21 signaling axes [7,8]. Notably, in AMD pathogenesis, RPE senescence triggers like amyloid-β aggregation and APOE-rich drusen formation impair lipid metabolism and retinal homeostasis [9,10,11]. Therapeutically, senescent RPE targeting shows promise, evidenced by the dual antioxidant and anti-senescence activity of nicotinamide [12,13] and MDM2-p53 inhibitor Nutlin-3a ameliorated retinal degeneration [14].

Dual-specificity phosphatase 4 (DUSP4), a multifunctional regulator of MAP kinase signaling, exhibits antioxidant and anti-inflammatory capacities [15,16]. Elevated DUSP4 expression in endothelial cells decreases oxidant-induced death and tissue injury [17,18]. DUSP4 regulates the survival and death of hepatocellular carcinoma cells by dephosphorylating ERK, JNK, and p38 MAPK [19]. Knockout of DUSP4 using CRISPR/Cas9 in N2A cell lines and organotypic suprachiasmatic nucleus slices enhanced vasoactive intestinal peptide responses through modulation of the ERK pathway [20]. Mechanistically, while wild-type DUSP4 exhibits substrate specificity for p-TBK1, the phosphatase-dead DUSP4 mutant completely abolishes this dephosphorylation capacity, confirming the enzyme’s catalytic dependency on its active site configuration [21]. A growing body of literature suggests that DUSP4 displays rather broad efficacy through the inactivation of ERK and p38-MAPK and the selective inhibition of JNK to regulate cell inflammation, immunity, and apoptosis.

Despite these pleiotropic functions, the role of DUSP4 in ocular pathophysiology remains underexplored. Our preliminary findings reveal significant DUSP4 dysregulation in AMD pathogenesis with strong correlations to senescence biomarkers. This study systematically investigates DUSP4’s functional contributions to AMD progression, particularly its regulatory effects on RPE senescence mechanisms. Our work aims to establish DUSP4 as a potential therapeutic target for modulating cellular senescence in dry AMD.

## 2. Results

### 2.1. The Upregulation of DUSP4 in Dry AMD

To investigate the involvement of DUSP4 in AMD, we first analyzed transcriptome profiles from the RPE–choroid complex in a clinical cohort (GSE29801) consisting of 31 normal, 26 AMD, and 11 potential pre-AMD donor eyes [22]. The AMD specimens were stratified into six distinct classifications: pre-AMD, sub-clinical pre-AMD, dry AMD, geographic atrophy (GA), choroidal neovascularization (CNV), and mixed GA/CNV. Notably, our reanalysis revealed significant upregulation of DUSP4 expression in dry AMD patients compared to controls.

**Figure 1:**
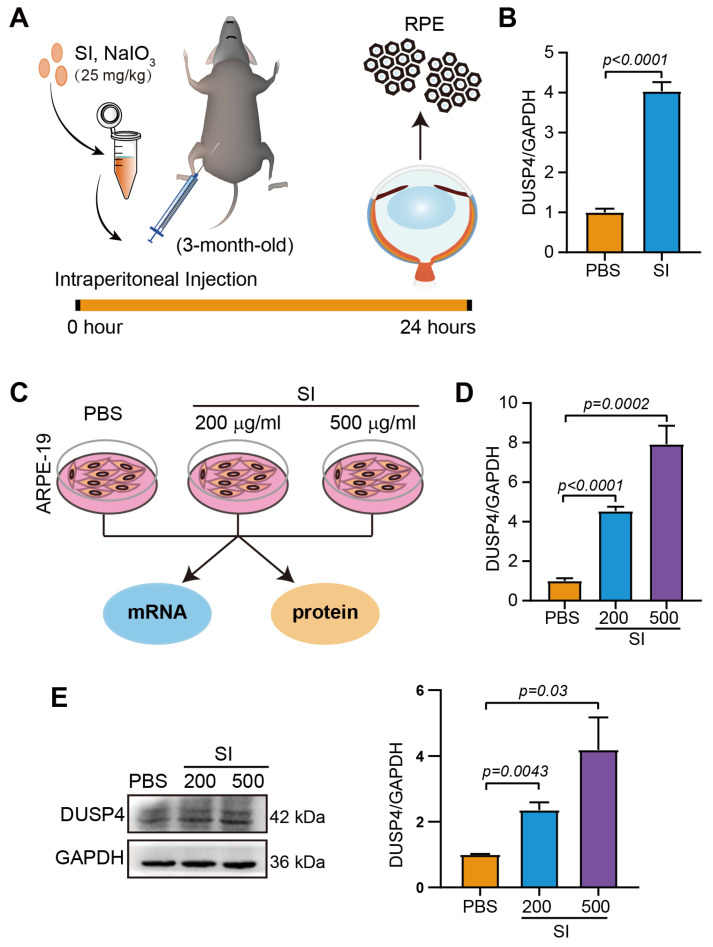
The expression of DUSP4 in RPE of dry AMD model. (**A**). Diagram illustrating SI-induced RPE damage in mice. (**B**). The expression of DUSP4 in the RPE of mice with SI injection for 24 h (*n* = 3). (**C**). Schematic diagram of ARPE-19 cells exposed to SI. (**D**). The mRNA expression of DUSP4 in ARPE-19 cells treated with SI (200 μg/mL; 500 μg/mL) for 24 h. (**E**). The protein expression of DUSP4 in ARPE-19 cells treated with SI at concentrations of 200 µg/mL or 500 µg/mL for 24 h. Band density was analyzed using ImageJ.

To validate this observation in an experimental model, we employed a well-established in vivo model, sodium iodate (SI)-induced retinal degeneration, which recapitulates key features of dry AMD through selective oxidative damage to RPE cells. Following intraperitoneal administration of SI, we observed progressive RPE cell loss over time (Figure 1A). Quantitative analysis of surviving RPE cells harvested 24 h post injection demonstrated significant elevation of DUSP4 mRNA levels compared to untreated controls (Figure 1B). Given the limited availability of primary murine RPE cells for protein-level analyses, we extended our investigation to the human ARPE-19 cell line. Cells exposed to SI (200 μg/mL or 500 μg/mL) for 24 h showed dose-dependent upregulation of DUSP4 expression (Figure 1C). DUSP4 was upregulated significantly at the mRNA level, particularly in the 500 μg/mL treatment group (Figure 1D). Consistent with these findings at protein levels, a 4-fold increase in DUSP4 expression was observed in high-dose-treated cells compared to controls (Figure 1E). These findings suggest that DUSP4 upregulation constitutes an adaptive response to oxidative stress in AMD pathogenesis.

### 2.2. Senescence of the RPE Is Accompanied by High Expression of DUSP4

**Figure 2:**
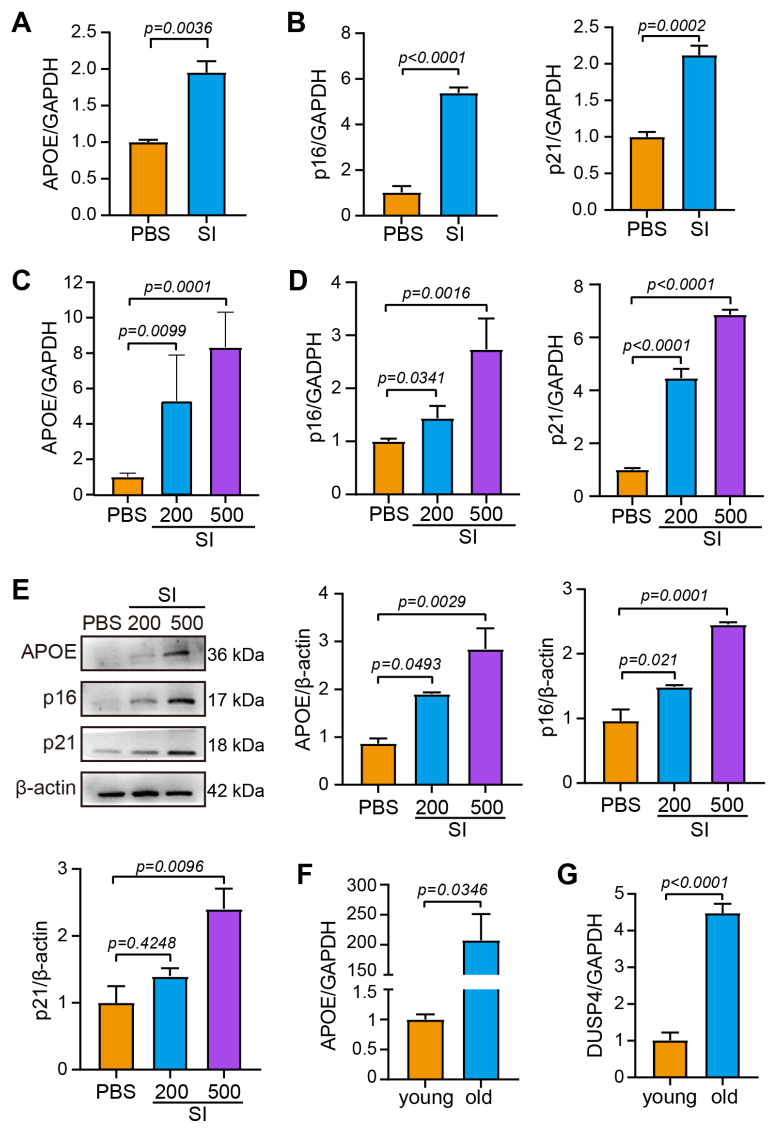
RPE senescence correlates with elevated DUSP4 expression. (**A**). APOE expression in mouse RPE 24 h after SI injection. (**B**). p16 and p21 expression in mouse RPE 24 h after SI injection. (**C**). APOE expression in ARPE-19 cells exposed to SI for 24 h. (**D**). p16 and p21 expression in ARPE-19 cells treated with SI for 24 h. (**E**). Protein expression of APOE, p16, and p21 in ARPE-19 cells 24 h after SI treatment. Band density was analyzed using ImageJ 1.48. (**F**). qPCR-quantified APOE expression in RPE from both young (10-week-old, *n* = 3) and old mice (15-month-old, *n* = 3). (**G**). qPCR-quantified DUSP4 expression in RPE of young and old mice.

To investigate RPE cellular senescence as a potential driver of AMD progression, we systematically analyzed canonical senescence markers p16 and p21 across AMD models. In the SI-induced murine AMD model, pathological validation was confirmed by significant elevation of the AMD-associated biomarker APOE (Figure 2A). Notably, RPE-specific upregulation of both p16 and p21 was observed in dry AMD model tissues (Figure 2B). This senescence phenotype was recapitulated in vitro using oxidative stress-challenged ARPE-19 cells, which exhibited dose-dependent increases in APOE (Figure 2C), p16, and p21 expression at transcriptional (Figure 2D) and protein levels (Figure 2E). These collective findings establish a causal link between AMD progression and RPE senescence exacerbation.

To mechanistically interrogate whether DUSP4 involved in AMD intersects with age-dependent RPE senescence, we conducted comparative analyses of RPE isolates from young (10-week-ole) versus aged (15-month-ole) mice. Compared with that in young mice, aged RPE cells displayed a dramatic elevation in APOE levels—a signature aligning with both senescence and AMD pathology (Figure 2F)—and the expression of DUSP4 was also upregulated more than four-fold (Figure 2G). This age-correlated co-upregulation of DUSP4 and senescence biomarkers strongly implicates DUSP4 as a molecular nexus bridging RPE aging processes and AMD development.

### 2.3. DUSP4 Deficiency in RPE Exacerbates AMD Progression

To understand the role of DUSP4 in the RPE, we generated DUSP4_KD in the RPE of mice via subretinal injection of AAV2/8-control shRNA-GFP or AAV2/8-mDUSP4 shRNA-GFP (Figure S1A). Fourteen days after injection, fundus imaging confirmed widespread green fluorescence in both control and DUSP4_KD groups, indicating successful viral transduction (Figure S1B). Frozen eyecup sections revealed GFP expression predominantly localized to the RPE layer, suggesting that the DUSP4_KD occurred mainly in RPE cells (Figure S1C). Immunofluorescence (IF) staining with an anti-DUSP4 antibody further demonstrated a marked reduction in DUSP4 expression within the RPE layer of DUSP4_KD mice compared to controls (Figure S1D). To quantify knockdown efficiency, RPE sheets were isolated, and qPCR analysis confirmed a >50% reduction in DUSP4 mRNA levels in the DUSP4_KD group (Figure S1E).

To assess the impact of DUSP4_KD on retinal structure and function, OCT and IF staining of ZO-1 were performed to detect retinal structure, and ERG was performed to detected visual function. OCT analysis revealed no significant differences in the thicknesses of the retina, outer nuclear layer (ONL), or RPE/photoreceptor between the control and DUSP4_KD groups (Figure S2A). Similarly, IF staining of ZO-1 showed no apparent alterations in RPE morphology or structure integrity in DUSP4_KD mice (Figure S2B). However, ERG recordings indicated a modest but consistent reduction in both a-wave and b-wave amplitudes in DUSP4_KD mice, suggesting a subtle impairment in visual function despite preserved retinal structure (Figure S2C).

**Figure 3:**
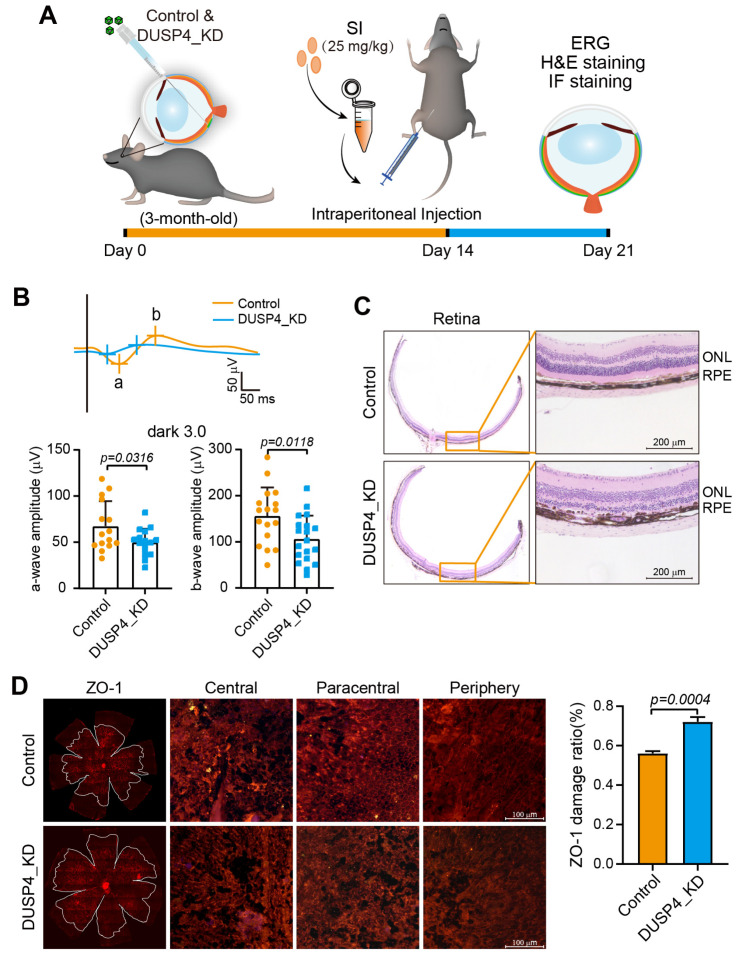
DUSP4 knockdown correlates with severe visual impairment in dry AMD mice model. (**A**). Schematic of an SI-induced dry AMD model with DUSP4 knockdown in the RPE. (**B**). Visual function comparison between DUSP4_KD and control groups in AMD mice: DUSP4_KD (*n* = 19) and control (*n* = 17). (**C**). Retinal structural damage in DUSP4_KD and control groups: DUSP4_KD (*n* = 6) and control (*n* = 6). (**D**). ZO-1 staining of RPE/choroid flat mount showed the damage of RPE in both DUSP4_KD and control groups: DUSP4_KD (*n* = 5) and control (*n* = 5). The proportions of damage areas were calculated by ImageJ 1.48. Statistical significance was determined by using the unpaired *t*-test.

To evaluate the role of DUSP4 in the progression of AMD, a low dose of SI was injected intraperitoneally into DUSP4_KD mice (Figure 3A). A visual function test revealed significantly lower a-wave and b-wave amplitudes in DUSP4_KD mice compared to controls, indicating exacerbated visual dysfunction (Figure 3B). Histological examination of retinal sections demonstrated more severe retinal damage in DUSP4_KD mice, particularly within the RPE layer, which exhibited increased melanin granule accumulation (Figure 3C). Further analysis of RPE integrity via IF staining of ZO-1 revealed pronounced disruption of the characteristic hexagonal RPE morphology in DUSP4_KD mice, with damage ratios of approximately 70% in the central and paracentral regions compared to 50% in controls (Figure 3D). Collectively, these findings suggest that DUSP4 deficiency exacerbates both structural and functional retinal damage in the progression of AMD.

### 2.4. DUSP4 Dysfunction Exacerbates RPE Senescence

Given the challenges of isolating sufficient primary RPE cells from mice for mechanistic studies, we employed ARPE-19 cells to establish in vitro models of DUSP4 dysfunction. Using CRISPR/Cas9 technology with single- or dual-restriction enzyme systems, we generated DUSP4_KO-1 and DUSP4_KO-2, two stable knockout cell lines targeting distinct functional domains of DUSP4. Then, 35 bp and 36 bp deletions in sister chromatids at the catalytic domain were deleted in the stable cell line DUSP4_KO-1. DUSP4_KO-2 inserted 43 bp into one allele and 20 bp into the complementary allele at the substrate-binding domain (Figure S3).

**Figure 4:**
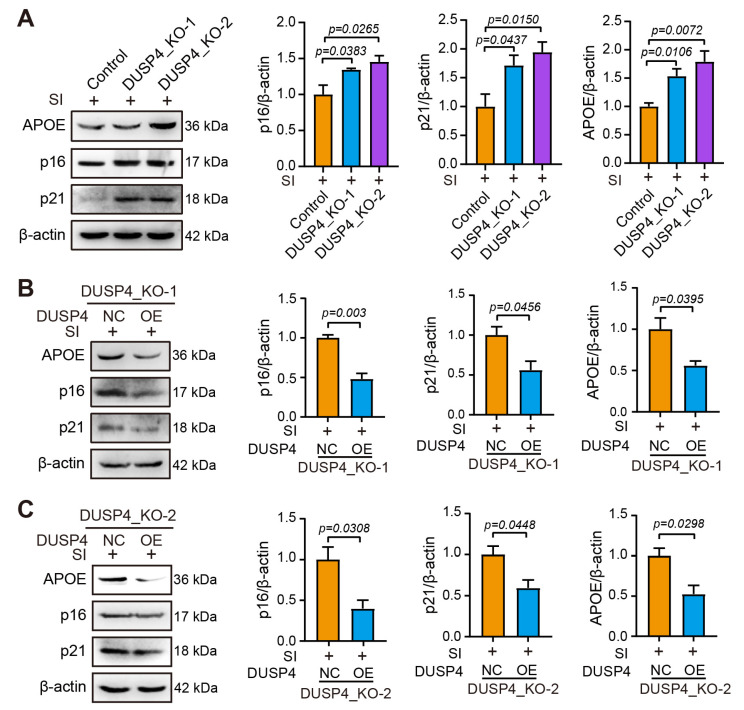
DUSP4 depletion enhances senescence marker expression in RPE. (**A**). DUSP4 knockout upregulates APOE, p16, and p21 expression in ARPE-19 cells. (**B**). DUSP4 overexpression reverses senescence marker upregulation in DUSP4_KO-1 cells. (**C**). DUSP4 overexpression reverses senescence marker upregulation in DUSP4_KO-2 cells. Band density was quantified using ImageJ 1.48. Statistical significance was determined by using the unpaired *t*-test.

SI treatment significantly upregulated APOE protein levels in both DUSP4_KO lines compared to controls, indicating enhanced susceptibility to AMD-like pathology. Concurrent increases in senescence markers p16 and p21 further confirmed accelerated cellular aging in DUSP4-deficient RPE cells (Figure 4A). To verify that the increased expression of senescence markers was caused by the lack of DUSP4, the functional DUSP4 was imported into both DUSP4_KO cell lines via plasmid transfection (Figure S4). Reconstitution of functional DUSP4 significantly attenuated SI-induced APOE accumulation, indicating restored protection against AMD-associated pathology. Quantitative analysis revealed concurrent reductions in senescence markers p16 and p21 in rescued DUSP4_KO-1 cells (Figure 4B). This phenotypic rescue was consistently replicated in DUSP4_KO-2 cells (Figure 4C), confirming the essential role of DUSP4 in mitigating RPE degeneration.

### 2.5. DUSP4 Slows RPE Senescence by Coordinately Regulating p38, p53, and NF-κB Pathways

**Figure 5:**
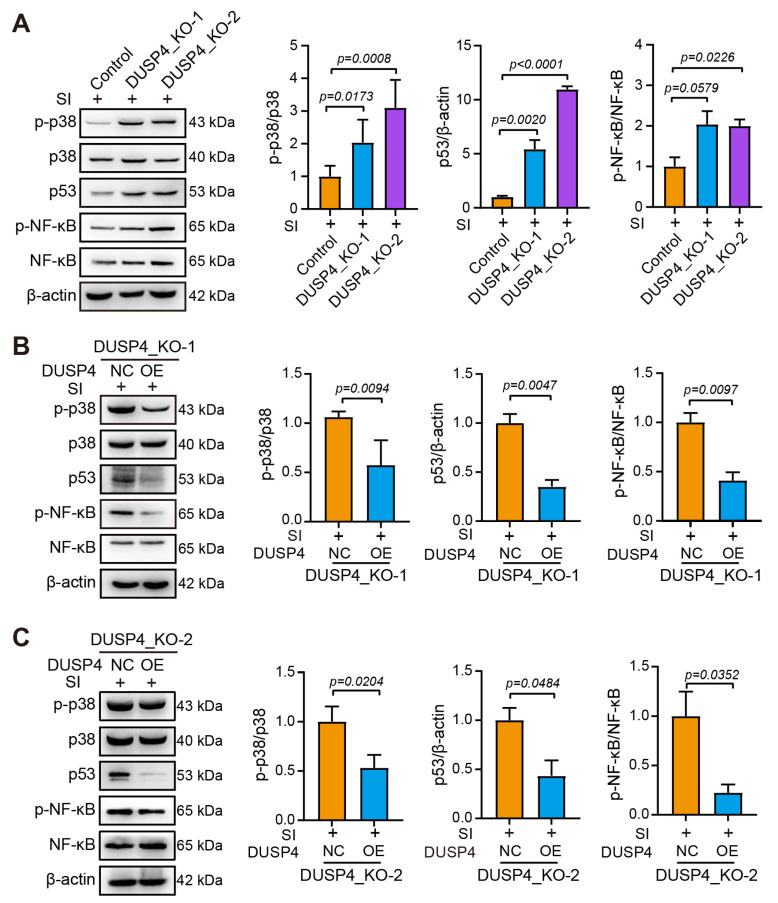
DUSP4 downregulated the p38, p53, and NF-κB signaling pathways. (**A**). p-p38, p53, and p-NF-κB expression in DUSP4_KO cell lines. (**B**). DUSP4 overexpression attenuated p-p38, p53, and p-NF-κB expression in DUSP4_KO-1 cells. (**C**). DUSP4 overexpression attenuated p-p38, p53, and p-NF-κB expression in DUSP4_KO-2 cells. Band density was quantified using ImageJ 1.48. Statistical significance was determined by using the unpaired *t*-test.

To elucidate how DUSP4 modulates senescence marker expression in SI-treated RPE cells, we interrogated three core signaling axes: p38 MAPK, p53/p21, and NF-κB. SI treatment in DUSP4_KO-1 and KO-2 cells robustly increased phosphorylated p38 (p-p38), a key mediator of stress-induced senescence that is normally inactivated by DUSP4-mediated dephosphorylation; SI exposure elevated p53 protein levels in both DUSP4_KO lines, driving p21 upregulation and subsequent cell cycle arrest; activated NF-κB (p-NF-κB Ser536) was markedly increased in DUSP4_KO lines, potentiating SASP and chronic inflammation (Figure 5A). Reintroduction of functional DUSP4 into DUSP4_KO-1 and KO-2 cells reversed these effects: p-p38 and p53 accumulation and NF-κB activation were all significantly attenuated (Figure 5B,C). The coordinated suppression of these pathways collectively reduced p16/p21 expression, mitigated SASP-related inflammation, and preserved RPE homeostasis.

## 3. Discussion

The present study unveils a previously unrecognized role of DUSP4 in suppressing RPE senescence during AMD pathogenesis. Our findings demonstrate that DUSP4 expression is upregulated in both clinical AMD specimens and SI-induced experimental models, where it mitigates RPE senescence through coordinated inhibition of p38, p53, and NF-κB signaling pathways. These results establish DUSP4 as a central adaptive regulator countering senescence-associated stress in AMD and underscore its therapeutic potential for targeting degenerative pathways.

The observed upregulation of DUSP4 in aged RPE and AMD models indicates a protective adaptation against senescence-associated damage. While chronic oxidative stress and inflammation are pathological hallmarks of AMD, the induction of DUSP4—a phosphatase with dual antioxidant and anti-inflammatory functions—likely reflects an endogenous attempt to rebalance cellular homeostasis. Nevertheless, this compensatory mechanism appears inadequate to fully halt AMD progression.

Mechanistically, the interplay between DUSP4 and senescence-associated markers p16 and p21 and regulators p38, p53, and NF-κB provides critical insights. The activation of p38 promotes inflammation, lipid accumulation, senescence, apoptosis, differentiation, and growth [23,24,25]. Notably, DUSP4 attenuates p38-driven inflammation and senescence by dephosphorylating activated p38 in RPE. p53 blocks cell proliferation [26]. Through p53/p21 axis inhibition, DUSP4 prevents p21-dependent cell cycle arrest in RPE. NF-κB is activated to promote cellular senescence via chronic inflammation [27,28]. DUSP4 further mitigates senescence-associated SASP induction by blocking NF-κB-mediated inflammation. Furthermore, the observed reduction in NF-κB activation upon DUSP4 overexpression highlights its role in attenuating SASP, a key contributor to the proinflammatory milieu in AMD.

The identification of p38, p53, and NF-κB as downstream effectors of DUSP4 offers further avenues for therapeutic intervention. Targeting these pathways in conjunction with DUSP4 modulation may enhance the efficacy of AMD treatments. The upregulation of DUSP4 in response to senescent stress during AMD highlights its potential as a therapeutic target. Enhancing DUSP4 expression or activity could provide a novel strategy to slow AMD progression by reducing oxidative stress and RPE senescence. Given the lack of effective treatments for dry AMD, targeting DUSP4 could address both oxidative stress and senescence—two interconnected drivers of disease progression. Notably, the efficacy of nicotinamide in AMD models [13] suggests that combining DUSP4 activation with p53 inhibitors or antioxidant agents might yield synergistic benefits.

While our study establishes the role of DUSP4 in RPE senescence, several questions remain. First, the SI-induced AMD model, while widely used, does not fully recapitulate the chronic, multifactorial nature of human AMD. Validating these findings in Ccl2/Cx3cr1 knockout genetic models or with alternative stressors would strengthen clinical relevance. Second, ARPE-19 cells, though convenient, exhibit differences from primary RPE in metabolism and senescence responses. Future work should confirm these results in patient-derived RPE or organoid models. Finally, the precise molecular interactions between DUSP4 and its downstream targets warrant further investigation using phosphor-proteomics or co-immunoprecipitation approaches.

## 4. Materials and Methods

### 4.1. Antibodies, Plasmids and Chemicals

The following antibodies were used: rabbit anti-DUSP4 (Merck, SAB4500677, St. Louis, MO, USA); rabbit anti-ZO-1 (Thermo Fisher Scientific, 617300, Waltham, MA, USA); rabbit anti-p21 Cip1 antibody (GeneTex, GTX27960, Irvine, CA, USA); rabbit anti-CDKN2A/p16INK4a antibody-N-terminal (Abcam, AB189034, Waltham, MA, USA); rabbit anti-p38 (Cell Signaling Technology, 8690, Danvers, MA, USA); rabbit anti-phospho-p38 (Cell Signaling Technology, 4511); rabbit anti-NF-κB p65 (Cell Signaling Technology, 8242); rabbit anti-phospho-NF-κB p65 (Cell Signaling Technology, 3033); rabbit anti-p53 (PTM, PTM-6319, Chicago, IL, USA); mouse anti-APOE (PTM, PTM-5915); anti-GAPDH recombinant rabbit monoclonal antibody (HUABIO, ET1601-4, Hangzhou, China); anti-beta actin mouse monoclonal antibody (HUABIO, EM21002); and iFluor™ 594-conjugated goat anti-rabbit IgG polyclonal antibody (HUABIO, HA1122). Human DUSP4 overexpression plasmids and control plasmids (GeneCopoeia, EX-A0606-M98, Rockville, MD, USA) were used in this study. AAV2/8-mDUSP4 shRNA-GFP and AAV2/8-control shRNA-GFP were purchased from Hanbio Biotechnology Company (Shanghai, China). The CRISPR/Cas9 plasmid systems, including the single-vector lentiCRISPRv2 (#52961) and the two-vector system comprising lentiGuide-puro (#52963) and sgRNA-lentiCas9n (D10A)-blast (#63593), were obtained from Addgene (Watertown, MA, USA). SI was purchased from Aladdin (7681-55-2; Riverside, CA, USA).

### 4.2. Animals

C57BL/6J mice of different ages were purchased from Charles River Laboratories (Beijing, China). Male and female mice were used in almost equal proportions. The mice were housed at the Animal Center of Wenzhou Medical University and maintained on a 12 h light/12 h dark cycle. The mice were anesthetized with a combination of pentobarbital sodium and xylazine at a weight of 0.008 mL/kg before subretinal injection or in vivo fundus photography. During anesthesia, the eyes were lubricated with a levofloxacin eye gel immediately after the investigations, and the mice were placed on a warming plate until they moved spontaneously, after which they were transferred to their cages. All experimental procedures were performed according to the guidelines of the ARVO Statement for the Use of Animals in Ophthalmic and Vision Research and were approved by the Wenzhou Medical University Animal Policy and Welfare Committee (wydw2022-0113).

### 4.3. Cell Culture and Transfection

The classic human RPE cell line used in the laboratory, ARPE-19, was purchased from ATCC (CRL-2302, Manassas, VA, USA). The cells were maintained in Dulbecco’s Modified Eagle Medium (DMEM)/F12 (Gibco, 11320033, Grand Island, NE, USA) supplemented with 10% fetal bovine serum (HyClone, SH30396.03, Marlborough, MA, USA), and 100 units/mL penicillin and streptomycin (Gibco, 15140122) in a humidified environment at 37 °C with 5% CO_2_.

For transfection, the cells were seeded into 6-well plates the day before transfection. DUSP4 overexpression plasmids were transfected with a Lipofectamine 3000 Transfection Kit (Thermo Fisher Scientific, L3000015) according to the manufacturer’s instructions.

### 4.4. DUSP4_Knockout (KO) Stable Cell Lines Edited by CRISPR/Cas9 Technology

DUSP4_KO sgRNAs were designed at two sites: the enzyme active site (sgRNA1: CCAGGCGGGCATCTCGCGGTCGG) and the substrate binding site (sgRNA2: AGCGG-CAGCCACGGCACCCTGG; sgRNA3: CGTTCCTGGCGCACAGCGCGG). The sgRNA constructs were strategically subcloned into distinct vector configurations: sgRNA1 was engineered into a single-vector system, while sgRNA2 and sgRNA3 were co-incorporated into a dual-vector expression platform. The plasmids were then transfected into ARPE-19 cells, which were selected with blasticidin or puromycin to create stable cell lines with editing of the DUSP4 gene. The edited DNA sites from stable cell lines were sequenced. Two stable cell lines from two different vectors, DUSP4_KO-1 and DUSP4_KO-2, both of which lack phosphatase activity, were selected for subsequent studies.

### 4.5. SI-Induced Dry AMD Model

Three-month-old C57BL/6J mice received an intravenous injection of SI solution at 25 mg/kg (body weight) to establish a dry AMD model. The same aged mice in the control group were injected with an equivalent dose of PBS. After 7 days, the fundus, visual performance, and retinal structure were detected.

### 4.6. Immunofluorescent (IF) Staining of ZO-1

After seven days of SI injection, the eyes were fixed in 4% paraformaldehyde for 1 h. The neuronal retina was detached, and each choroid/RPE/sclera tissue sample was cut with four radial incisions. The RPE–choroid–sclera complex eyecups were washed three times with PBS. Choroidal flat mounts were blocked with 10% bovine serum albumin (Solarbio, A8020, Beijing, China) and 0.5% Triton X-100 (Beyotime, P0096, Shanghai, China) for 2 h at room temperature. After that, they were incubated with ZO-1 antibodies at 4 °C overnight. The choroidal flat mounts were then washed thoroughly with PBS and incubated with iFluor™ 594-conjugated goat anti-rabbit IgG at room temperature for 2 h. Finally, an anti-fluorescence quenching agent (Beyotime, P0131) containing 4,6-diamino-2-phenyl indole (DAPI) was used to seal the sections. The images were captured under an orthotopic fluorography microscope (Leica, Wetzlar, Germany) with an excitation wavelength of 594 nm.

### 4.7. DUSP4_KD in the RPE of Mice

The AAV-shDUSP4 was purchased from HANBIO and designed shRNA sequence: top strand: GATCCGAAAGGCTGATGAACCGGGATGAGAATTCAAGAGATTCTC ATCCCGGTTCATCAGCCTTTTTTTTTC; bottom strand: AATTGAAAAAAAAAGGCT GATGAACCGGGATGAGAATCTCTTGAATTCTCATCCCGGTTCATCAGCCTTTCG. The knockdown of DUSP4 in the RPE was performed by subretinal injection of AAV-shDUSP4. Subretinal injection mainly targets RPE and photoreceptor cells. Three-month-old C57BL/6J mice were anesthetized via an intraperitoneal injection of pentobarbital/xylazine mixture, and their pupils were dilated via tropicamide phenylephrine eye drops. Subretinal injection was performed under a surgical microscope (Leica). An insulin needle (0.33 mm × 12.7 mm) was used to make a scleral puncture approximately 1 mm behind the corneoscleral margin. Then, a cover glass (24 mm × 24 mm) was placed over the cornea to help visualize the retina directly. The head of the Hamilton needle (Bio-Strategy, V Australia) was then gently guided through the conjunctiva, sclera, and retina slowly into the vitreous cavity, bypassing the crystal body, until it reached the posterior pole of the opposite retina. Next, 2 μL of AAV2/8-mDUSP4 shRNA-GFP (1.4 × 10^12^ μg/mL) or AAV2/8-control shRNA-GFP was injected into one eye of each mouse. Any problems with the injection, including large backflow upon removal of the needle and hemorrhaging of vessels, were noted, and those eyes were excluded from the analysis. After 14 days, green fluorescence spread throughout the fundus, and the DUSP4_KD model was constructed.

### 4.8. Optical Coherence Tomography (OCT) and Fundus Imaging

After anesthesia, the mice were administered a proper amount of tropicamide eye drops to dilate their eyes, and ofloxacin eye cream was applied to their corneal surface to prevent infection and enhance the coupling between the front lens and the eye surface. The mice were then placed on the Micro IV stage (Phoenix, AZ, USA), with their eyeballs facing the front lens. Using a fundus retinal imaging system, the visual nipple image was exactly at the center of the visual field. An OCT imaging acquisition system was used to obtain images of the retina.

### 4.9. Electroretinography (ERG)

The mice were dilated with tropicamide and anesthetized with sodium pentobarbital after 8 h of acclimation to the dark. The corneal surface of the mouse eye was then anesthetized with proparacaine hydrochloride, and the pupils were dilated with tropicamide. Beneath the dark red light, the mice were positioned on the operating table. Circular corneal electrodes were placed on the mouse’s bilateral corneal surface, and a needle stainless steel reference electrode needle was inserted beneath the skin of both sides of the gills. Furthermore, the needle ground electrode was penetrated under the mouse tail. After baseline stabilization, a-wave and b-wave amplitude changes for dark-adapted 3.0 and light-adapted 3.0 ERGs were recorded.

### 4.10. Quantitative Real-Time Polymerase Chain Reaction (qPCR)

To isolate RNA from the ARPE-19, DUSP4_KO-1, and DUSP4_KO-2 cells, TRIZOL (Beyotime, R0016) was used following the manufacturer’s instructions. The isolated RNA was then reverse-transcribed into cDNA via the HiScript III All-in-one RT SuperMix Perfect for qPCR (Vazyme, R333, Nanjing, China). Taq Pro Universal SYBR qPCR Master Mix (Vazyme, Q712) was used for RT-PCR. Tsingke (Beijing, China) provided the required primers, the sequences of which are listed as follows: APOE-homo-F:CTGGCACTGGGT CGCTTTTG, APOE-homo-R: GGATGGGGACACTCACCTCAG; APOE-mus-F: GCA AGTCCTTGCTCCATACCT, APOE-mus-R: AAGACAATTTTTCCCTCCGCA; p16-homo-F: CTTCGGCTGACTGGCTGG, p16-homo-R: TCATCATGACCTGGATCGGC; p16-mus-F:GAGTCCGCTGCAGACAGACT, p16-mus-R:CCAGGCATCGCGCACATCC A; p21-homo-F: TGTCCGTCAGAACCCATGC, p21-homo-R: AAAGTCGAAGTTCCA TCGCTC; p21-mus-F: GCCCGAGAACGGTGGAACTT, p21-mus-R: GACAAGGCCACG TGGTCCTC; DUSP4-homo-F: ATGCCGTGAAGGACTGCCGTG, DUSP4-homo-R: TGG CATATTGATCACAGACGA; DUSP4-mus-F: CCCGTCGAAGACAACCACAA, DUSP4-mus-R: AACGCCTAAAATCCCCACCA; and GAPDH-m/h-F: GAACGGGAAGCTCACTGG, GAPDH-m/h-R: GCCTGCTTCACCACCTTCT. Finally, the expression level of each gene was estimated and normalized to that of GAPDH.

### 4.11. Western Blotting

Total protein was recovered from cell lysates in the presence of protease inhibitors (BOSTER, AR1182-1, Wuhan, China). Protein concentrations were determined with a BCA protein assay kit (Yeasen, 20201ES, Shanghai, China). The protein samples were denatured in a hot water bath before being resolved on a 10–12% gradient gel via sodium dodecyl sulfate–polyacrylamide gel electrophoresis at 80 V for 90 min and then 220 mA for 60 min. The separated proteins were transferred to polyvinylidene difluoride (PVDF) membranes (Bio-Rad, 1620177, Philadelphia, PA, USA). First, the membranes were blocked with 5% dried skim milk (Beyotime, P0216) in 1× Tris-buffered saline (Aladdin, T431531)-0.1% Tween 20 (Beyotime, ST1726). Next, primary antibodies were added, and the mixture was gently agitated for 2 h at room temperature or overnight at 4 °C. DUSP4, p16, p21, APOE, p53, p38, p-p38, NF-κB, and p-NF-κB expression was analyzed using previously described antibodies, with b-actin or GAPDH serving as an internal control. Finally, images were captured with a ChemiDox XRS imaging system (Bio-Rad) using an enhanced chemiluminescence (ECL) immunoblot kit. ImageJ was used to analyze the band density.

### 4.12. Retinal Histology

Retinal histology was performed via hematoxylin and eosin (H&E) staining. The eyes were removed from the mice and prepared for histopathology. A cross-sectional slice of the retina was chosen from each eye that contained the optic nerve head area. The paraffin slice thickness was set at 5 μm. Images were taken from the same part of the retina layer via a digital inverted fluorescence microscope (Leica) at 20× magnification. The total retina thickness was measured via ImageJ 1.48 software.

### 4.13. Statistical Analysis

The data are presented as the means ± SEMs of at least three independent experiments and were analyzed via GraphPad Prism 10.1.2 (GraphPad Software, San Diego, CA, USA). The differences between the two groups were analyzed via *t*-test. The difference was considered significant when *p* < 0.05.

## 5. Conclusions

This study identified DUSP4 as a pivotal regulator of RPE senescence in AMD, bridging oxidative stress, inflammation, and cell cycle arrest. Its ability to modulate p38, p53, and NF-κB pathways positions DUSP4 at a nexus of senescence signaling, offering a multi-faceted therapeutic target. While challenges remain in translating these findings to clinical interventions, our work underscores the potential of senescence-targeted therapies to alter the trajectory of dry AMD, a disease long deemed intractable. Future research should focus on optimizing DUSP4 activation strategies and exploring combinatorial approaches to enhance RPE resilience in aging and disease.

## Data Availability

The original contributions presented in this study are included in the article/Supplementary Materials. Further inquiries can be directed to the corresponding authors.

## Supplementary Materials

The following supporting information can be downloaded at https://www.mdpi.com/article/10.3390/ijms26083735/s1.

## Abbreviations

The following abbreviations are used in this manuscript:

DUSP4	Dual-specificity phosphatase 4
AMD	Age-related macular degeneration
SI	Sodium iodate
APOE	Apolipoprotein E
p16	p16INK4a
p21	p21Cip/WAF1
ERG	Electroretinography
H&E	Hematoxylin and eosin
IF	Immunofluorescence
ZO-1	Zonula occludens-1
KO	Knockout
KD	Knockdown
GFP	Green fluorescent protein
