# Supplementary material for: The Protective Role of DUSP4 in Retinal Pigment Epithelium Senescence and Degeneration
Source: Int J Mol Sci. 2025 Apr 15;26(8):3735. doi: 10.3390/ijms26083735 (PMC12027498; doi:10.3390/ijms26083735)
Supplement: Supplementary file 1 [file ijms-26-03735-s001.zip › ijms-3557404-supplementary.pdf]

International Journal of
Molecular Sciences

MDPI

Article

The protective role of DUSP4 in retinal pigment epithelium senescence and degeneration

Supplement Figures

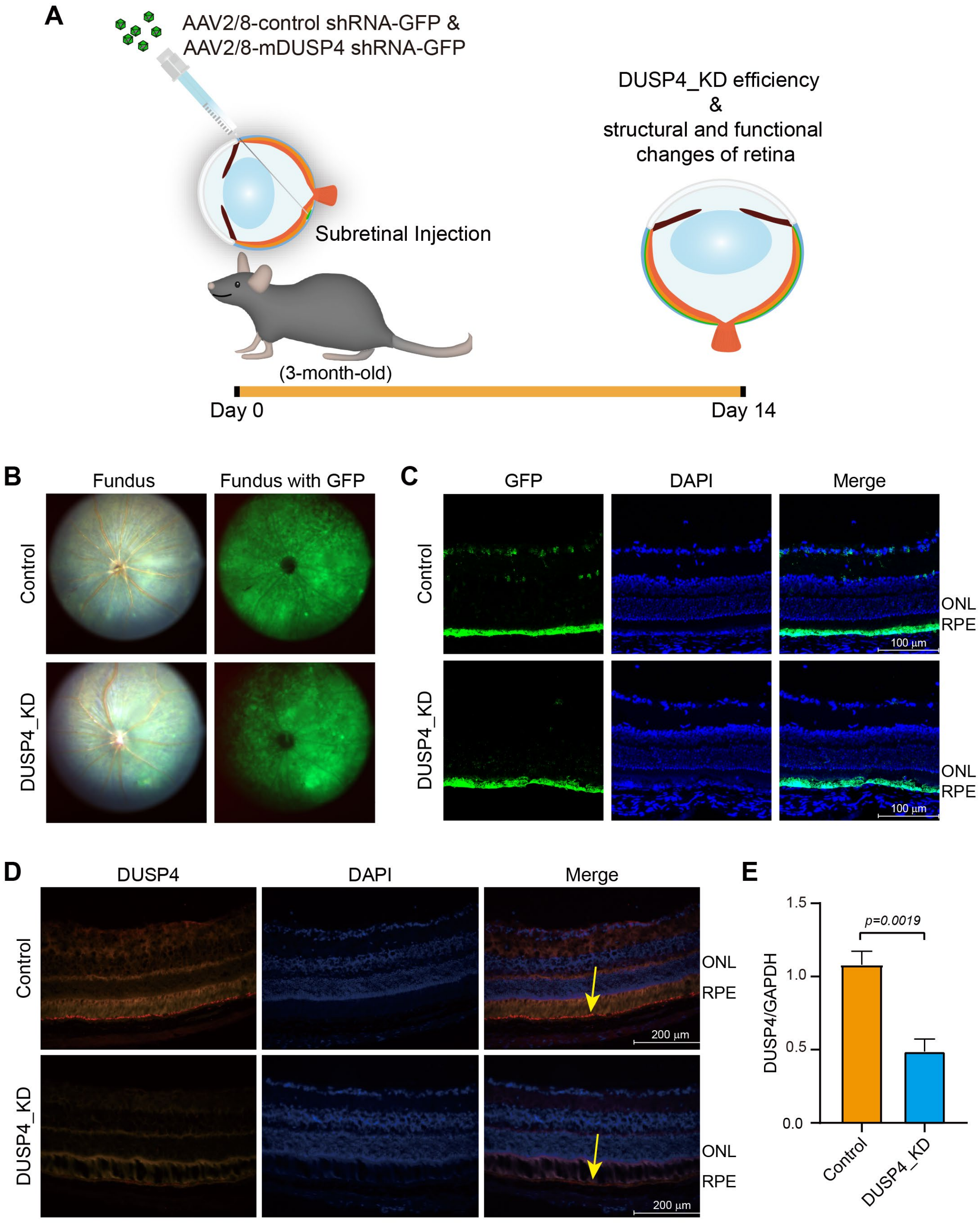

 https://doi.org/10.3390/ijms26083735

Supplementary Figure S1. The knockdown of DUSP4 in the RPE of mice. A. Diagrammatic drawing of DUSP4 knockdown in the RPE of mice by subretinal injection of AAV-shRNA. B. Bright-field and fluorescence imaging of fundus in DUSP4_KD and control groups, DUSP4_KD (n=4), control (n=4). C. Retinal frozen sections showed the distribution of control shRNA-GFP or mDUSP4 shRNA-GFP after subretinal injection. DUSP4_KD (n=6), control (n=6). D. IF staining confirmed the knockdown of DUSP4 in RPE layer of the DUSP4_KD and the control groups, DUSP4_KD (n=3), control (n=3). E. qPCR detected the efficiency of DUSP4 knockdown in RPE layer, DUSP4_KD (n=3), control (n=3).

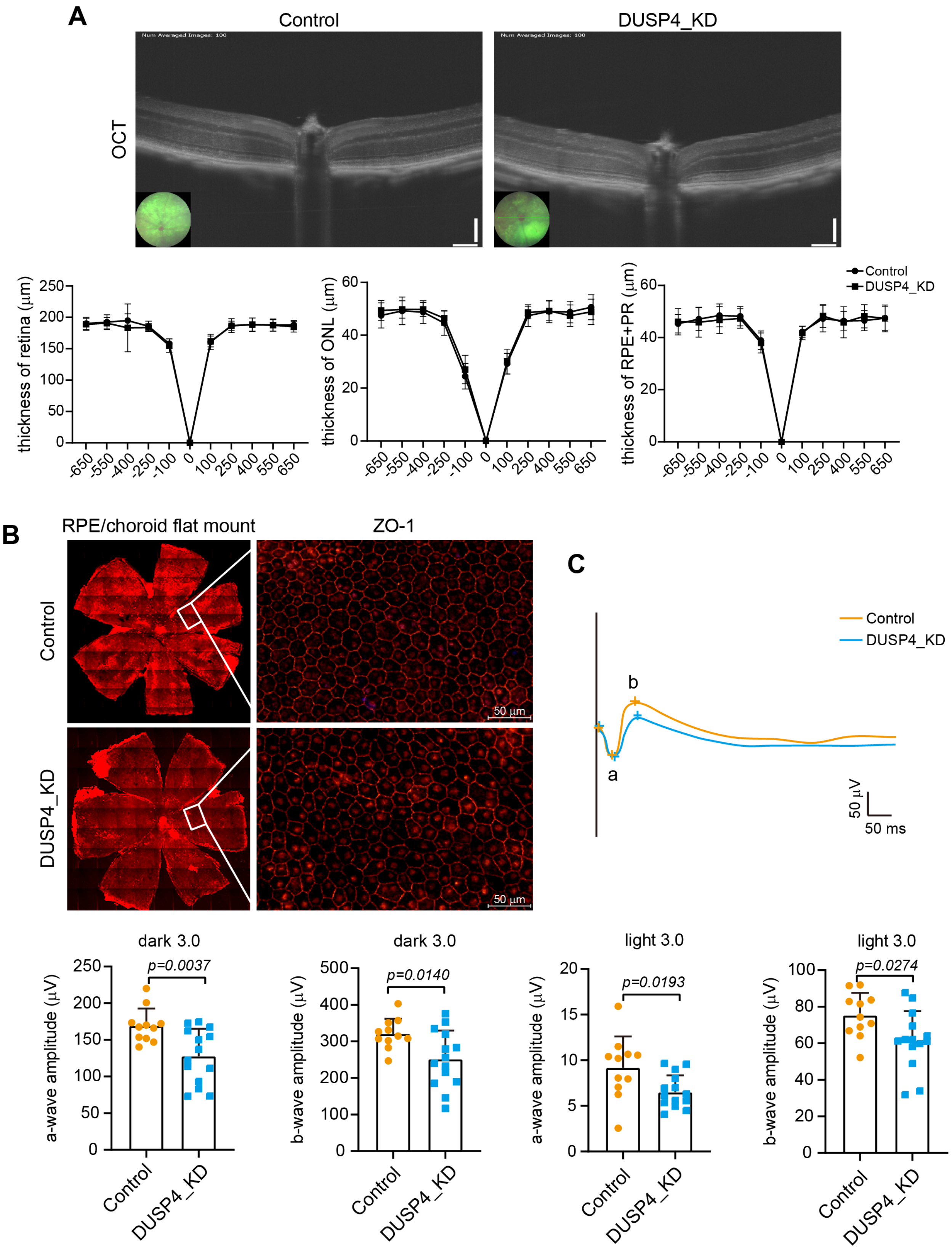

Supplementary Figure S2. Effects of DUSP4 knockdown on visual function and retinal structure. A. OCT detected retinal structure of the DUSP4_KD and control groups, DUSP4_KD (n=10), control (n=10). B. ZO-1 staining showed the RPE morphology in DUSP4_KD and control groups, DUSP4_KD (n=3), control (n=3). C. ERG detected visual function of the DUSP4_KD and the control groups, control (n=11), DUSP4_KD (n=14). Statistical significance was determined by using the unpaired t-test.

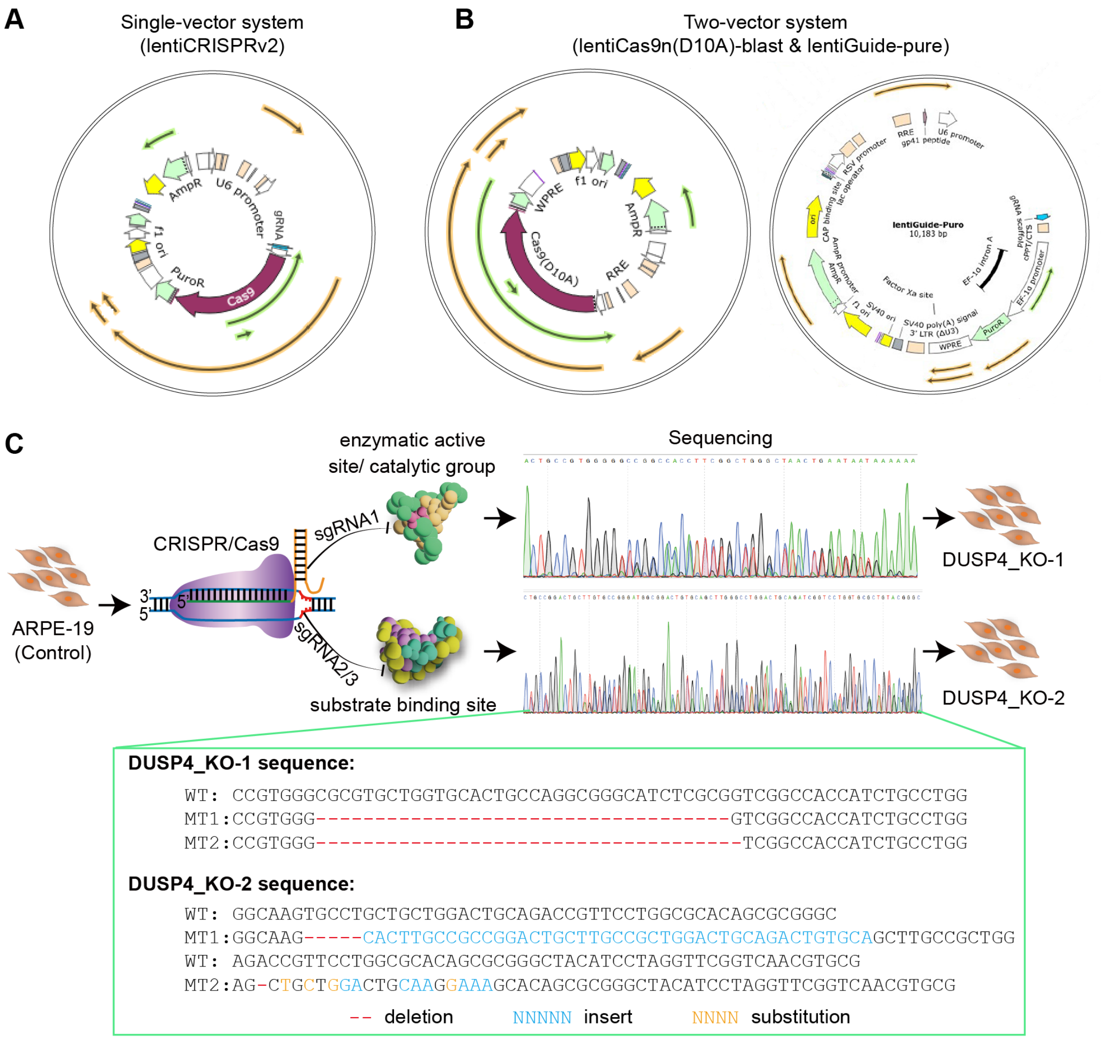

Supplementary Figure S3. Generation of stable DUSP4_KO cell lines via CRISPR/Cas9 technology. A. CRISPR/Cas9 single-vector system plasmid map. B. CRISPR/Cas9 two-vector system plasmid maps. C. sgRNAs were cloned into both single-vector system and two-vector system. The modifiy plasmids were then transfected into ARPE-19 cells, followed by selection with puromycin or blasticidin. Stable cell lines were generated by sequencing of the target locus.

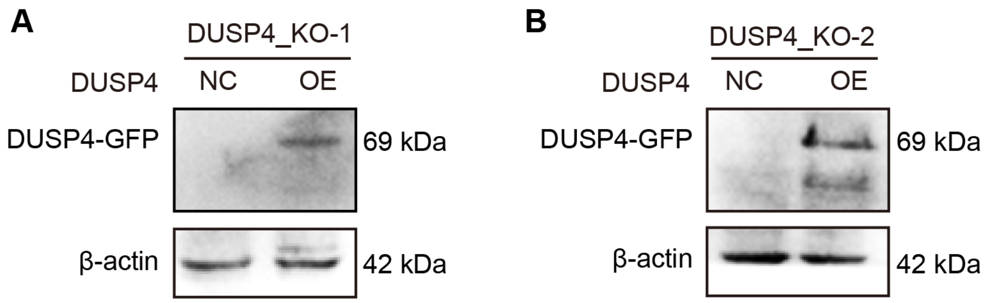

Supplementary Figure S4. DUSP4 overexpression in the DUSP4_KO cell lines. A. The overexpression of DUSP4 in the DUSP4_KO-1 cell line. B. The overexpression of DUSP4 in the DUSP4_KO-2 cell line.

Disclaimer/Publisher's Note: The statements, opinions and data contained in all publications are solely those of the individual author(s) and contributor(s) and not of MDPI and/or the editor(s). MDPI and/or the editor(s) disclaim responsibility for any injury to people or property resulting from any ideas, methods, instructions or products referred to in the content.
